# Antenatal pyelonephritis: a three-year retrospective cohort study of two Irish maternity centres

**DOI:** 10.1007/s10096-023-04609-6

**Published:** 2023-05-01

**Authors:** Rachel Barry, Elaine Houlihan, Susan J. Knowles, Maeve Eogan, Richard J. Drew

**Affiliations:** 1 Department of Clinical Microbiology, Rotunda Hospital, Dublin, Ireland; 2 Department of Clinical Microbiology, National Maternity Hospital, Dublin, Ireland; 3 Women’s and Children’s Health, University College Dublin, Dublin, Ireland; 4 Department of Obstetrics and Gynaecology, Rotunda Hospital, Dublin, Ireland; 5 Clinical Innovation Unit, Rotunda Hospital, Dublin, Ireland; 6 Irish Meningitis and Sepsis Reference Laboratory, Children’s Health Ireland at Temple Street, Dublin, Ireland; 7 Department of Clinical Microbiology, Royal College of Surgeons in Ireland, Dublin, Ireland

**Keywords:** Antenatal pyelonephritis, Urinary tract infection, Infections in pregnancy

## Abstract

Pyelonephritis affects 1–2% of pregnant women, and is associated with significant maternal and fetal morbidity. Antenatal pyelonephritis has been associated with PPROM (preterm premature rupture of membranes), preterm labour, low birth weight (LBW) and prematurity. A three-year retrospective dual-centre cohort study of antenatal pyelonephritis cases was conducted in two neighbouring Irish maternity hospitals – the Rotunda Hospital (RH) and the National Maternity Hospital (NMH). Patient demographics, clinical presentation, investigations, management and maternal/neonatal outcomes were recorded. A total of 47,676 deliveries (24,768 RH; 22,908 NMH) were assessed. 158 cases of antenatal pyelonephritis were identified (*n* = 88 RH, *n* = 70 NMH), with an incidence of 0.33%. The median age was 28 years. The median gestation was 27 + 6 weeks, with 51% presenting before 28 weeks’ gestation. Risk factors included; obesity (18.4%), diabetes mellitus (13.3%) and self-reported clinical history of recurrent urinary tract infection (28.5%). Rate of relapse with UTI in the same pregnancy was 8.2%. Renal ultrasound was performed in 30.4%. Predominant uropathogens were *Escherichia coli* (60%), *Klebsiella pneumoniae* (11%) and *Proteus mirabilis* (5%). 7.5% of cases had a concurrent bloodstream infection, 13.3% of cases were complicated by sepsis and 1.9% with septic shock. Complications including PPROM (6.3%), preterm delivery < 37 weeks’ gestation (11%), LBW < 2,500 g (8.2%) were comparable between sites. Delivery within 72 hours of diagnosis was noted in 7% (*n* = 11) of patients, of which three were preterm and one had LBW. Appropriate and prompt investigation and management of antenatal pyelonephritis is essential given the associated maternal and neonatal morbidity.

## Introduction

Urinary tract infections (UTIs) are the most common bacterial infection to occur during pregnancy and are classified as either involving the lower urinary tract (cystitis) or the upper urinary tract (pyelonephritis). Physiological and hormonal changes during pregnancy can lead to a higher frequency of asymptomatic bacteriuria, occurring in 2–15% of parous women [1]. Approximately 90% of pregnant women develop ureteral dilatation which typically begins in week 6 of pregnancy, peaks during weeks 22 to 24, and persists until the time of delivery. Urinary stasis and ureterovesical reflux occur secondary to increased bladder volume and decreased bladder and ureteral tone. Other contributing factors include; increased plasma volume, development of glycosuria encouraging bacterial growth and increased levels of urinary progesterone and oestrogen which causes smooth muscle relaxation [1]. Although pyelonephritis only affects 1–2% of pregnant women, it is associated with significant maternal and fetal morbidity [2]. Antenatal pyelonephritis has been associated with PPROM (preterm premature rupture of membranes), preterm labour (defined as labour before 37 weeks’ gestation) and low birth weight (LBW) infants (defined as birth weight < 2,500 g) [1]. Typical clinical manifestations include pyrexia, loin pain, dysuria, rigors and/or clinical signs of sepsis, similar to non-pregnant adults. Untreated pyelonephritis may lead to ascending infection and progression to systemic sepsis, with the additional risk of renal tract complications including abscess formation [3].

There are many risk factors for pyelonephritis including pregnancy-specific (nulliparity, younger maternal age) and non-pregnancy-specific factors (diabetes mellitus, urinary catheterisation, renal calculi, recurrent UTIs). Antenatal pyelonephritis is reportedly most common during the second half of pregnancy, as a result of physiological changes from the gravid uterus [4].

Enterobacterales are the predominant cause of UTIs both in pregnant and non-pregnant adults, with *Escherichia coli* representing the majority of pathogens, followed by *Proteus mirabilis* and *Klebsiella pneumoniae* [5]. Group B streptococcus (GBS) is a less common cause of UTI, however has important implications in pregnancy [6].

The mainstay of treatment for acute pyelonephritis is systemic antibiotics. National guidelines recommend parenteral treatment until the patient is 48 hours afebrile, followed by an appropriate oral switch to complete a ten-day treatment course (or seven-day if treating with a quinolone antibiotic) [3]. Shorter durations have proven effective in pyelonephritis [7], but data is limited in pregnant women. A randomised control trial which evaluated a modest sample of pregnant women who received initial intravenous therapy until 48 hours asymptomatic showed a 5.6% readmission rate to hospital when oral antibiotics were used to complete a 10 day course, versus a 12.9% readmission rate when no oral antibiotics were continued [8]. Ceftriaxone 1–2 g once daily intravenously is recommended as a first-line treatment for the treatment of antenatal pyelonephritis. Clindamycin or vancomycin (depending on the GBS susceptibility result) in addition to gentamicin is used in those with penicillin allergy as second-line [3].

The National Maternity Hospital (NMH) and the Rotunda Hospital (RH) represent two proximate large maternity centres, serving a similar catchment population in Dublin city centre, Ireland. Both hospitals deliver approximately 7,000—9,000 women per year, have an identical Electronic Health Record system and aligning antimicrobial prescribing guidelines.

Although several international guidelines exist, there is a paucity of recent epidemiological studies which examine predictors and outcomes of antenatal pyelonephritis. This retrospective cohort study aims to address this research gap by identifying confirmed cases of antenatal pyelonephritis over a three-year period and assessing risk factors, clinical course, management and materno-fetal outcomes, thus adding to the current knowledge-base and creating a greater understanding of the natural history of this disease.

## Methods

A three-year (2018–2020) retrospective review was undertaken to identify all women with antenatal pyelonephritis in NMH and RH. Data from 2021 was excluded due to an unauthorised cyber-attack which was launched by criminals upon Irish hospitals in May 2021, resulting in a lapse in electronic documentation during this period.

A search was conducted via the Electronic Patient Record (MN-CMS, Maternal and Newborn Clinical Management System, Cerner) which captured all antenatal women who were admitted for parenteral antibiotics to either maternity hospital from 2018–2020 inclusive. The antibiotics were chosen based on the recommended agents for pyelonephritis in each centre’s Antimicrobial Prescribing Guidelines, and included: ceftriaxone, cefotaxime, meropenem, ciprofloxacin, aztreonam, and vancomycin. Gentamicin was excluded given that it is not recommended as a single agent treatment for pyelonephritis, and we felt its addition would inappropriately broaden the search.

An electronic chart review was then performed on all generated patients to establish if they fit the IDSA (Infectious Diseases Society of America) consensus definition for pyelonephritis [9]. For the purposes of this review, symptoms compatible with diagnosis included fever, rigors, chills, nausea, vomiting or costovertebral angle tenderness (including back or flank pain). If inclusion criteria were met, a more detailed electronic chart review was undertaken. Data collected included information on baseline demographics, clinical presentation, investigations, microbiological findings, delivery and maternal/neonatal outcomes.

‘Sepsis’ and ‘septic shock’ were defined as per the National Clinical Guidelines on sepsis management [10]. Early intervention in the management of sepsis is based on the “Sepsis Six” approach which includes taking bloods (including lactate), blood cultures, monitoring urine output, administration of appropriate intravenous fluids (crystalloid), antibiotics as per local policies and supplemental oxygen as required. “Sepsis Six + 1” is used in antenatal patients, with the addition of fetal monitoring [10].

Further demographic data was collected from the ‘Annual Clinical Activity and Management Reports’ 2018–2020, which are published annually by each maternity hospital and are accessible via the hospital websites.

Personal data was irrevocably anonymised and stored in a password encrypted excel file in compliance with the General Data Protection Regulation legislation (2016). Statistics were performed using XLSTAT. Data was presented in tables using Chi-squared tests and *p*-values to describe differences between variables. Statistical significance was defined as a *p *value of < 0.05.

## Results

Over the three-year study period, there were a total of 47,676 deliveries between the two centres (24,768 RH, 22,908 NMH). Comparative clinical activity from the 2018–2020 annual reports is summarised in Table 1.

**Table 1:** Comparison of annual clinical activity reports 2018–2020

	RH	NMH	*p* value
Total mothers delivered > 500 g	24,768	22,908	
Total births > 500 g	25,240	23,325	
Antepartum deaths per total births > 500 g (% of all births)	0.4%	0.39%	0.73
LBW (< 2500 g) per total births > 500 g (% of all births)	6.45%	5.68%	**< 0.004**
Preterm per total births > 500 g	7.3%	7.0%	0.21
Mode of delivery: SVD	47–50%	55.9–57.2%	
Mode of delivery: operative vaginal delivery	16–17%	12.5–13.7%	
Mode of delivery: CS	34–37%	28.9–31.4%	
Perinatal mortality rate	5.4–7.7%	4.3–9.2%	

Items in bold represent values deemed statistically significant (*p* value <0.05)

Key: LBW = low birth weight, SVD = spontaneous vaginal delivery, CS = Caesarean section

The initial search for antenatal patients who were admitted for intravenous antibiotics from 2018–2020 identified 504 patients (247 in RH and 257 in NMH). Of those, a total of 158 cases met the IDSA definition of pyelonephritis and were included in the review (*n* = 88 RH, *n* = 70 NMH).

Cases of antenatal pyelonephritis are summarised in Table 2. The median gestation at presentation was 27^+6^ weeks (range 5^+4^—41^+4^), with more first/second trimester cases of antenatal pyelonephritis in RH (58%) than in NMH (41%) (*p* = 0.039). The median age at presentation was 28 years (range 15–48), with patients in RH, on average, younger than those in NMH (27 years versus 31 years). Parity of patients ranged from 0 to 7, with median parity of 0. Nulliparous women represented 53% of patients (*n* = 84), and the distribution of nulliparous versus multiparous was similar between sites. The median BMI (body mass index) in this cohort was 25 kg/m^2^, which is categorised as overweight (25-30 kg/m^2^), and the overall rate of obesity (BMI > 30 kg/m^2^) was 18.4%. A self-reported clinical history of recurrent UTI was documented in 28.5% of patients (*n* = 45), with a significantly higher rate in RH (35%) versus NMH (20%) (*p* = 0.035). Documentation of diabetes mellitus (13.3%) was comparable between sites. Relapse of UTI within the same pregnancy (defined as presentation to the maternity centre and/or readmission with positive urine culture and symptoms compatible with UTI) was noted in 8.2%. Antimicrobial prophylaxis following an episode of pyelonephritis was documented in only 5% (*n* = 8) of patients (*n* = 7 nitrofurantoin, *n* = 1 unknown). Renal ultrasound was performed in 30.4% (*n* = 48) of cases, of which 14.5% (*n* = 7) showed conclusive evidence of infection. There was no statistically significant difference in performance of renal ultrasound between hospitals *(p* = 0.67).

**Table 2:** Summary of cases of antenatal pyelonephritis

		Total cases	RH	NMH	*p* value
Cases pyelonephritis	Total	158	88 (56%)	70 (44%)	
	1st trimester (< 13 weeks)	13	7	6	
	2nd trimester (13–27^+6^ weeks)	65	43	22	
	3rd trimester (≥ 28 weeks)	80	38	42	
Median gestation at presentation		27^+6^ (5^+4^- 41^+4^)	26^+4^	30^+4^	
Maternal factors					
Median age at presentation		28 (15–48)	26	31	
Median parity		1 (0–7)	1	1	
	Nulliparous	84 (53%)	48	36	0.697
	Multiparous	74 (47%)	40	34	0.697
Median BMI (kg/m^2^)		25 (17–48)	26	25	
Obesity (> / = 30 kg/m^2^)		29 (18.4%)	12	17	
Diabetes mellitus (*n* =)		21 (13.3%)	13	8	0.473
History of recurrent UTI (*n* =)		45 (28.5%)	31	14	**0.035**
Investigations					
Renal US performed (*n* =)		48 (30.4%)	24	24	
	Inpatient	41	24	17	0.670
	Outpatient	7	0	7	**0.002**
Clinical features					
Bloodstream infection (*n* =)		12 (7.5%)	2	10	
Sepsis (*n* =)		21 (13.3%)	10	11	0.424
	Septic shock	3 (1.9%)	3	0	0.119
HDU/ICU admission (*n* =)		12 (7.6%)	7	5	0.848
UTI relapse (same pregnancy) (*n* =)		13 (8.2%)	9	4	0.305
Renal US (*n* =)	Total performed	48 (30.4%)	24	24	
	Pyelonephritis	6 (12.5%)	6	0	
	Abscess	1 (2%)	0	1	
	Hydronephrosis	26 (54%)	11	15	
	Renal stone	1 (2%)	1	0	
	No significant findings	14 (29%)	6	8	
Management					
Median duration of IV antibiotics (days)		3 (1–11)	3	3	
Median duration of total antibiotics (days)		10 (2–16)	9	10	
Median length of stay (days)		4 (1–18)	3	4	
Delivery					
Mode of delivery (*n* =)	SVD	70 (44.3%)	39	31	
	Instrumental delivery	22 (13.9%)	12	10	
	LSCS	65 (41.1%)	36	29	
	Other (ERPC)	1 (0.6%)	1	0	
Pregnancy loss (< 24 weeks) (*n* =)		1 (0.6%)	1	0	
Intrauterine death (> 24 weeks) (*n* =)		1 (0.6%)	1	0	
PPROM (*n* =)		11 (7.0%)	7	4	0.347
Preterm delivery (*n* =)		18 (11%)	13	5	0.21
	Extremely preterm < 28 weeks	1	1	0	
	Very preterm 28–32 weeks	2	2	0	
	Moderate-late preterm 32–37 weeks	15	10	5	
Infant factors					
Median birth weight (kg)		3.3 (0.96–4.4)	3.3	3.5	
Low birth weight (< 2500 g) (*n* =)		13 (8.2%)	11	2	0.057
	ELBW < 1000 g	1	1	0	
	VLBW 1000-1500 g	1	1	0	
	LBW 1500-2500 g	11	9	2	

Items in bold represent values deemed statistically significant (*p* value <0.05)

Key: BMI = body mass index, UTI = urinary tract infection, US = ultrasound, HDU = high dependency unit, ICU = intensive care unit, IV = intravenous, SVD = spontaneous vaginal delivery, LSCS = lower segment Caesarean section, ERPC = evacuation of retained products of conception, PPROM = preterm premature rupture of membranes, ELBW = extremely low birth weight, VLBW = very low birth weight, LBW = low birth weight

Twenty-one people (13.3%) had a diagnosis of sepsis during the episode of pyelonephritis with three (1.9%) of these patients requiring management for septic shock. The most common SIRS criteria met were; tachycardia (heart rate ≥ 100 beats per minute) in 95% (*n* = 20), pyrexia (temperature ≥ 38 or ≤ 36 degrees celcius) in 81% (*n* = 17) and tachypnoea (respiratory rate ≥ 20 breaths per minute) in 47% (*n* = 10). Completion of “Sepsis Six + One” was satisfactory in the majority of cases, with 100% of patients having blood cultures drawn, as well as receiving appropriate intravenous fluids and systemic antibiotics.

Of the total 158 patients with antenatal pyelonephritis, eleven patients delivered within 72 hours of clinical presentation (*n* = 6 RH, *n* = 5 NMH). Three of these patients delivered preterm (< 37 weeks). The first patient had PPROM resulting in spontaneous delivery at 36^+3^ weeks. The second patient was induced at 36^+3^ weeks (instrumental delivery) in the setting of sepsis with relapsed pyelonephritis requiring HDU admission. The final patient was delivered by emergency Caesarean section (CS) at 33^+3^ weeks due to complex maternal co-morbidities, delivering a low birth weight baby (2130 g). The other ten infants were of normal birth weight. Five required admission to the NICU (neonatal intensive care unit) and there were no fatalities in this group.

The predominant organism isolated from urine was *Escherichia coli* (*n* = 110, 60%), followed by *Klebsiella pneumoniae* (*n* = 17, 11%) and *Proteus mirabilis* (*n* = 8, 5%). Drug-resistance was noted in 6.4% (*n* = 10), with five cases of ESBL (extended spectrum betalactamase), four cases of *AmpC* betalactamase, and one *Klebsiella oxytoca* with an unclassified resistance phenotype. Twelve people (7.5%) had concurrent bloodstream infection during the episode of pyelonephritis, of which five were complicated by sepsis, without septic shock. *E. coli* represented the majority of bloodstream infections at 83%, followed by *K. pneumoniae*. Two patients had pyelonephritis secondary to Group B streptococcus (*S. agalactiae*), neither of which were complicated by PPROM or early onset neonatal sepsis.

Appropriate antibiotics were chosen, with 83% (*n* = 131) of women treated with ceftriaxone, in line with local and national antimicrobial guidelines. Meropenem was the selected agent in 14% of cases (*n* = 22) which is the treatment of choice in the setting of a clinically deteriorating patient, septic shock, or in a patient in whom an MDRO (multi-drug resistant organism) is suspected. Meropenem was mostly used in the RH group (*n* = 19). This increased use of meropenem in RH was likely attributable to a recently observed increase in the rate of ESBL (66% increase in 2018–2020 when compared to the preceding 3-year period). Antibiotics were de-escalated to a more narrow spectrum agent where possible, when antimicrobial susceptibility results were available. The average duration of intravenous antibiotics (3 days) and total antibiotics (9 days) was considered appropriate for treatment of antenatal pyelonephritis, and the average length of inpatient hospital stay was 4 days; all comparable between sites.

The majority of women (44%, *n* = 70) had unassisted vaginal births, followed by 41% by LSCS (lower segment Caesarean section) (*n* = 65), and 14% instrumental (*n* = 22). One case of late miscarriage at 18 weeks was delivered by ERPC (evacuation of retained products of conception).

Rates of PPROM, preterm delivery and LBW were comparable between sites.

PPROM was noted in 6.3% (*n* = 10) cases. Preterm delivery was noted in 11% (*n* = 18) of cases, of which 3 (1.9%) were directly related to antenatal pyelonephritis. The majority (56%, *n* = 10) of these pregnancies were delivered intentionally early (7 LSCS, 3 inductions) due to maternal and fetal factors. A total of 8 women (44%) had unassisted vaginal births. The majority of preterm births (83%, *n* = 15) subclassified as “moderate to late preterm” with delivery between 32 and 37 weeks’ gestation. There were two cases of “very preterm birth” (28–32 weeks), and one case of “extreme prematurity” (< 28 weeks), all in the RH group.

The median birth weight was 3,300 g. Overall, there were thirteen babies with low birth weight (LBW), the majority of whom (85%) weighed in the moderate LBW bracket (1,500 g-2,500 g). Two outliers in RH were identified: The first baby was ‘very low birth weight’ (< 1,500 g) delivered by SVD with PPROM at 26 weeks, resulting in a complicated clinical course and neonatal death at 8 weeks of life. The second was ‘extremely low birth weight’ (< 1000 g), delivered by SVD with PPROM at 29 weeks.

Neonatal death was recorded in 4 cases, all of which were unrelated to antenatal pyelonephritis.

## Discussion

The incidence of antenatal pyelonephritis in this dual-centre retrospective cohort study was 0.33%, which is lower than that of other published reports (1–2%). The majority of cases (78%) occurred in the second half of pregnancy (> 20 weeks’ gestation) in keeping with the current literature [4, 11]. Interestingly, nulliparous women were underrepresented in our cohort (53%) compared to previous studies which quote up to 75% of cases affecting nulliparous women [12]. Of note, RH and NMH are exclusively maternity hospitals and it is possible that some women may have been cared for in general hospitals prior to fetal viability (for example, before their ‘booking visit’).

A BMI of > 25 kg/m^2^ (‘overweight’) was noted in 52% of cases, which is associated with a higher incidence of perinatal problems including congenital anomalies (specifically neural tube defects and cardiac defects), preeclampsia and infection [13]. Diabetes mellitus was documented in 13.3% (*n* = 14 gestational, *n* = 7 non-gestational) which further potentiates the risk of perinatal complications. However, this may be an underestimation as it was subject to documentation at the ‘booking visit’ by healthcare staff, prior to the gestation at which screening for gestational diabetes mellitus is usually performed (after week 20).

Relapse of UTI within the same pregnancy was noted in 8.2% of women, a rate much lower than that described in previous studies (up to 23%) [5]. Of note, this figure only captures patients who attended their maternity hospital with UTI, and will not include those who were treated in the community. National guidelines state that antimicrobial prophylaxis should be considered after an episode of antenatal pyelonephritis [3]. Nitrofurantoin is considered the first-line agent, however should be avoided near term or imminent delivery due to the risk of neonatal haemolysis. The decision to use antimicrobial prophylaxis was recorded in only 5% of patients, however, this was subject to documentation by medical staff at the time of discharge or at their follow up renal outpatient appointment. There are no clear evidence-based recommendations relating to the use of prophylaxis post antenatal pyelonephritis, and further research is required to extrapolate the risks versus benefits of this intervention. The predominant organism was *E. coli*, consistent with literature of pyelonephritis in pregnant and non-pregnant adults [3].

Delivery within 72 h of diagnosis with pyelonephritis occurred in 7% (*n* = 11) of patients, three of which resulted in preterm births and one of which resulted in a LBW infant, highlighting the morbidity and mortality associated with antenatal pyelonephritis. One could hypothesise that the diagnosis of pyelonephritis directly precipitated labour, or that there may have been a simultaneous diagnosis of chorioamnionitis in these cases.

The rate of preterm labour among cases of antenatal pyelonephritis was 11%, which is higher than that of the overall antenatal population in both centres (7–7.3% Table 1). Of note, the majority (83%) of preterm deliveries were between 32–37 weeks, which is associated with reduced neonatal morbidity compared with ‘very’ or ‘extremely preterm’ births [14]. Similarly, the rate of LBW infants (8.2%) was higher than the overall antenatal population (5.5–6.4% Table 1). However, 85% of these were between 1500-2500 g which is again associated with reduced neonatal morbidity compared with those < 1500 g [15]. It should also be noted that detailed comorbidities among cases of pyelonephritis were not recorded and thus, it is possible that our patients represent a disproportionately comorbid population given the propensity for infection leading to hospital admission, creating an inherent bias within the study with respect to maternal and neonatal outcomes.

## Conclusion

This, to our knowledge, is the first cohort study of antenatal pyelonephritis in Ireland. Despite broad search criteria (including all women who received intravenous antibiotics) and a considerable sample size of over 47 thousand women, the incidence of antenatal pyelonephritis as per the IDSA consensus definition was low at 0.33% of births. However, complications such as relapse of infection, sepsis, septic shock, multidrug resistance, preterm labour and LBW infants were all noted in our study, highlighting the significant morbidity and mortality associated with this condition. Prompt recognition and treatment with appropriate targeted antimicrobials and timely implementation of the Sepsis Six + One pathway should be encouraged, and should be a focus for education and future audit.

## Data Availability

The data that support the findings of this study are available from the corresponding author, RB, upon reasonable request.

## Code availability

N/A.
